# Canine-Assisted Speech Therapy for Children: Caregiver and Therapist Perspectives

**DOI:** 10.3390/healthcare13243299

**Published:** 2025-12-16

**Authors:** Zhao Yue Zhang, Carlie Driscoll, Jessica Hill, Tiffani Howell, Genevieve Ward

**Affiliations:** 1 School of Health & Rehabilitation Sciences, University of Queensland, Brisbane, QLD 4072, Australia; zhaoyue.zhang@uq.net.au (Z.Y.Z.); jessica.hill@uq.edu.au (J.H.); 2 School of Psychology & Public Health, La Trobe University, Bendigo, VIC 3552, Australia; t.howell@latrobe.edu.au; 3 Independent Researcher, Australia; gen-ward@hotmail.com

**Keywords:** animal-assisted therapy, canine-assisted paediatric speech pathology, canine-assisted speech pathology, communication disorders, speech pathology, speech therapy, therapy dogs

## Abstract

**Background/Objectives:** This study investigated the perceived impact of therapy dogs on speech therapy for children with communication disorders. **Methods:** Using an interpretive descriptive design, we gathered insights from both therapists and caregivers. Five Australian speech pathologists who incorporate therapy dogs into their practice participated in semi-structured interviews, and forty-two caregivers of children who received canine-assisted speech therapy completed online surveys. **Results:** Collectively, the responses revealed that therapy dogs helped children to establish rapport with the therapist, regulate their emotions and behaviours, engage with therapeutic tasks, be motivated to attend, use natural language, and improve social skills. The therapists reported personal and professional benefits, including enhanced job satisfaction and reduced stress, but highlighted the necessity of tailoring sessions to suit both the child and the dog, noting challenges in managing this dynamic. **Conclusions:** The study concluded that integrating therapy dogs into speech therapy is perceived to enhance therapeutic outcomes for children and improve therapist well-being. These findings offer valuable insights for incorporating therapy dogs into speech therapy practices, potentially improving the communication skills and quality of life of children with speech and language difficulties.

## 1. Introduction

Animal-assisted therapy (AAT) has emerged as a valuable addition in various therapeutic settings, leveraging the natural bond between humans and animals to facilitate rehabilitation and improvement in patients’ well-being [1]. Therapy animals can naturally capture people’s attention in a non-threatening manner, offering a calming effect. Their affectionate responses to human interaction can foster pro-social behaviour and positive emotions, serving as an emotional bridge to aid in therapeutic interactions [2]. For instance, AAT has been found to reduce behavioural and psychological symptoms of dementia in a variety of healthcare settings [3]. It has also been demonstrated as beneficial for autistic people, who often exhibit communication difficulties, limited social interaction, and behaviours of concern [4,5,6]. These characteristics make it more challenging to elicit engagement, communication, and interaction, which are crucial factors in successful therapeutic outcomes, particularly in fields such as Speech Pathology.

Among the diverse applications of AAT, canine-assisted therapy has garnered attention for its unique benefits. Dogs, with their innate ability to provide emotional support and foster social engagement, have shown promise in enhancing the therapeutic process for children with behavioural and developmental disorders, as reviewed by Hüsgen et al. [7]. Interaction with therapy dogs is perceived to increase engagement, motivation, behavioural regulation, comfort and confidence, enjoyment of therapy, as well as verbal and non-verbal communication in autistic children, thereby potentially improving therapeutic outcomes [8]. The inclusion of a therapy dog has also been suggested to ease children’s weariness and help them become familiar with the clinical setting much quicker, facilitated by the therapy dog’s non-judgmental demeanour [8].

To address communication impairments, a qualified speech pathologist works in conjunction with a specially trained therapy dog in each session to provide canine-assisted speech pathology services. To date, few studies have investigated such services for paediatric populations. Machová et al. [9] investigated the effects of canine-assisted speech therapy on motor and facial motricity skills in children with developmental dysphasia and found that, compared to the conventional one-on-one therapist to patient model, canine-assisted speech therapy sessions significantly improved these skills. Tanner and Macauley [10] also found positive outcomes for a sample of five preschoolers who received canine-assisted speech pathology for language delays. Finally, Siemons-Luhring [11] reported on memory skill enhancement in 22 children with language disorders, comparing performance with and without the presence of a therapy dog. Their results revealed significantly improved memory following canine-assisted speech therapy. Notably, these studies were focused on quantitative clinical outcomes, largely overlooking the relational and emotional aspects that are crucial for understanding the broader impact of canine-assisted therapy.

Despite its potential, there is also a deficit in real-world implementation studies surrounding AATs in the communication sciences. Most often, studies do not delve into the practical challenges or considerations that therapists face when incorporating a therapy dog into speech therapy sessions. From the therapist’s perspective, their focus often lies on the practical aspects and therapeutic efficacy of incorporating a therapy dog into therapy sessions, while also managing the challenges of balancing traditional therapeutic goals with the inclusion of an animal. Their insights can reveal the logistical considerations of AAT, such as maintaining focus, ensuring safety, and tailoring sessions to individual needs. Understanding how therapists integrate canine-assisted therapy into their existing frameworks is crucial for establishing practice guidelines and developing effective training programmes for professionals.

Furthermore, within a range of healthcare disciplines, although not yet including speech pathology, studies looking at the parents’ perspectives with regard to their children’s canine-assisted therapy sessions are becoming increasingly accessible (e.g., Hill et al.’s and London et al.’s studies in occupational therapy [8,12]). The caregiver’s unique role allows them to act both as an observer during therapy sessions and as an observer of the effects of the intervention in the child’s daily life—a perspective not accessible to the therapist. Understanding the experiences of those involved in canine-assisted therapy can highlight the benefits and potential challenges of integrating canine-assisted interventions into speech therapy, contributing to a deeper understanding of how such therapies can support the well-being and communication development of clients. Insights from these perspectives are essential for improving clinical practices and refining therapeutic approaches in canine-assisted speech therapy.

This study aimed to examine both caregiver and therapist perspectives on canine-assisted speech therapy for children. Together, these perspectives can provide a holistic view of how canine-assisted speech pathology is perceived to influence the therapeutic process and to enhance outcomes for children with communication disorders.

## 2. Methods

### 2.1. Study Design

This study employed a prospective interpretive descriptive design. Ethical clearance was provided by the University of Queensland Human Research Ethic Committee (2024/HE000231). All participating therapists were provided with a detailed project information sheet and gave verbal assent to participate in one-on-one interviews. Caregivers indicated electronic consent prior to accessing the study’s purposefully designed descriptive survey and voluntarily submitting their responses.

### 2.2. Participants and Setting

Purposeful sampling was used to recruit five Australian speech pathologists who were employed as independent contractors at a private speech pathology service located in the state of Victoria, Australia, to provide traditional and canine-assisted speech therapy to children. Recruitment was based on the therapists’ availability to participate in online Zoom interviews.

Measures were taken to mitigate potential bias from the clinic owner (GW), one of the therapists interviewed. The clinic owner was excluded from viewing and/or analysing any project data. In addition, all responses were anonymised.

The caregiver cohort consisted of 42 individuals whose children had participated in at least one canine-assisted speech therapy session at the clinic. All caregivers on the clinic’s client list were notified of the opportunity to participate through email communication from the lead researcher (Z.Y.Z.) and were invited to complete a structured online descriptive survey.

### 2.3. Data Collection and Analysis

The therapists participated in a single, individual, semi-structured interview each, a format chosen to provide both structure and flexibility, allowing for in-depth exploration of their perspectives while ensuring that key topics were consistently addressed across participants (see Appendix A for interview questions). This approach was deemed particularly suitable for this study, as it facilitated the collection of rich, qualitative data on therapists’ experiences and insights concerning canine-assisted therapy, while simultaneously allowing participants the freedom to elaborate on topics they considered most pertinent. Therapists booked their interview times by contacting the lead researcher (Z.Y.Z.), a final-year Master of Audiology Studies student at the University of Queensland, who conducted the interviews.

All interviews were audio-recorded to ensure accuracy in capturing the therapists’ responses, and these recordings were subsequently transcribed verbatim by the lead researcher (Z.Y.Z.). Interviews lasted between 24 min and 45 min. A total of 25 interview questions encompassed demographic information of the therapist and dog, operational factors, service delivery and client interaction, and the clinical impacts of incorporating therapy dogs. Therapists were invited to give open-ended answers to express anecdotal experiences, observations, and personal reflections regarding their work.

Caregivers were invited to share their perspectives on the canine-assisted therapy services their children received through an online survey, which consisted of 13 multiple-choice and open-ended questions (see Appendix B for survey questions). Like the interview questions for the therapists, the caregiver survey questions were specifically designed for this study and based upon similar projects conducted by authors JH and CD in canine-assisted occupational therapy, wherein the questions had been piloted and validated prior to use. The survey was administered through the Qualtrics platform, via a link accessible to all caregivers. It was designed to obtain a comprehensive understanding of caregivers’ perspectives, covering areas such as the perceived impact on their child’s communication skills, emotional well-being, and overall experience with canine-assisted speech therapy. Survey data were compiled and imported into IBM SPSS Statistics 29 software for descriptive analysis of demographic information.

Open-ended interview and survey data were analysed using reflexive thematic analysis according to the six-phase approach outlined by Braun and Clarke [13]. Data was familiarised through repeated readings of transcripts and survey responses, and initial codes were generated by systematically identifying notable features across the entire dataset. These codes were then collated into potential themes through patterns and relationships within the data. Coding and establishment of themes were performed by the first and second authors, independently. Themes were then collaboratively refined to ensure they accurately reflected the data and addressed the research questions, with each theme being defined and named to clearly capture its essence. Finally, a detailed report was produced, integrating the thematic findings with illustrative quotes from participants to provide a coherent narrative.

## 3. Results

### 3.1. Caregivers and Children

#### 3.1.1. Demographics

A total of 42 caregivers completed the study survey, providing their perspectives on the canine-assisted speech pathology service provided to their children. The mean age of children was 7 years 8 months (SD = 4 years 1 month, range = 2–19 years, *n* = 33). Males represented 55% of the sample (*n* = 23); females represented 45% (*n* = 19). Most children currently had or previously had a pet dog at home (*n* = 31/42; 73.8%). Most commonly, the children had been receiving canine-assisted therapy for 2 years or less (*n* = 36/42; 86%).

The children were most frequently attending therapy for treatment of speech delays (*n* = 20), language delays (*n* = 16), and speech disorders (*n* = 12). Refer to Table 1 for a complete listing of treated conditions. The children’s therapy goals varied broadly, with 54 individual areas of focus referred to by the caregivers. Most commonly, the goals concerned development of social skills (20/54; 37.0%), and speech development and production (14/54; 25.9%). Less frequently, goals were related to fluency (3/54; 5.6%), emotional regulation (2/54; 3.7%), and school-readiness (2/54; 3.7%). In response to a request for any other important information about their child, few replies were received, and several caregivers noted their child’s additional co-morbidities, such as ASD, ADHD, and hearing loss.

#### 3.1.2. Perspectives on Canine-Assisted Speech Pathology

As shown in Table 2, caregivers reported that their children interacted with the therapy dog during sessions in many different ways, both actively and passively, with the majority of children (>64%) engaging in physical touch with the dog, provision of a treat to the dog, playing with a toy or game together, talking to the dog, and watching the dog.

Most caregivers indicated that their children “enjoyed” the canine-assisted speech pathology sessions (*n* = 37; 88.1%). A small number of caregivers were “neutral” (*n* = 4; 9.5%) and one reported that their child “somewhat enjoyed” the sessions (*n* = 1; 2.4%). Additionally, most caregivers (>50%) perceived that the therapy dog had a positive influence on their children’s mood, stress and anxiety levels, engagement in activities, motivation, and engagement/rapport with the therapist. No caregivers reported any negative influences (see Table 3). Furthermore, caregivers perceived that the therapy dog’s presence influenced achievement of their children’s therapy goals in a variety of ways, with 54 individual areas of focus (data points) referred to by the caregivers. Most commonly, caregivers noted the effects of motivation and willingness to attend therapy (14/54), as well as provision of emotional support and calming (13/54). See Table 4 for full theme listing and supporting quotes.

Regarding a question on the difference between therapy sessions with and without the therapy dog present, caregivers’ responses contained a total of 38 semantic elements (data points). Predominantly, caregivers noted that their children were less engaged, focussed, and attentive during therapy when the dog was absent (16/38). They also reported their children to be more anxious, stressed, and in poorer mood without the presence of the therapy dog (6/38). Notably, 6 of 42 caregivers did not notice a difference in therapy based on dog presence versus dog absence.

Caregivers were also asked to describe their child’s relationship with the therapy dog. Thematic analysis of 43 individual semantic elements (data points) in caregivers’ responses revealed the most prevalent themes to be: Relationship Development (13/43; e.g., “Great relationship, loves the therapy dog,” and “Considers a friend.”), followed by Positive Emotional States (9/43; e.g., “Joyful, happy, friend, his buddy,” and “Curious, interested, and happy.”), with Calming Effects and Anticipation/Excitement tied (4/43 each; e.g., “Helps to calm his nervous energy” and “She just loves them and is so excited to attend”, respectively.). Minor themes included the enhancement of engagement in therapy sessions and the provision of comfort/safety for the child. Overall, the overwhelming sentiment in these comments was positive, suggesting therapy dogs create meaningful connections that enhance therapeutic experiences. However, one respondent noted an allergic skin reaction in their child during and after a canine-assisted session. It was unknown whether this child was prone to any other allergies.

Regarding any canine-assisted service improvements that caregivers could suggest, nearly all of the seven provided responses were positive and related to wanting “more,” for example, having the dog always present at sessions and with every clinician, additional dogs, or more involvement of the dog in sessions. One respondent commented that the ability to conduct therapy while walking the dog would be beneficial for their child.

Finally, in response to an open-ended question seeking any further comments, 19/42 (45%) caregivers chose to leave replies. The vast majority of comments reflected the theme of caregivers and children being very happy with the canine-assisted therapy experience (*n* = 12/19; 63%), for example: “I feel that having a therapy dog has been positive and beneficial to my child.” Others commented on the motivation provided by the presence of the dog in therapy sessions (*n* = 3/19; 2%); “a good motivator to encourage listening…” A few caregivers also mentioned the formation of a bond between child and dog, improved engagement in sessions, and a reduction in fear of animals.

### 3.2. Therapists

#### 3.2.1. Demographics of Therapists and Therapy Dogs

The five Speech Pathologists interviewed had between 1.5 and 8 years of overall experience in the profession (Median = 2.25 years), and between 1.25 and 4 years of specific experience in canine-assisted speech pathology (Median = 1.75 years). All therapists owned their therapy dogs, whom they had purchased from a breeder or adopted. The dogs were of varied breeds, including Weimaraners, Rottweilers, Groodles, and Golden Retrievers, and ranged in age from 1 to 14 years old. There was no particular gender pattern in the therapy dogs.

One of the five therapists had undergone specific training in animal-assisted therapy, both formal (through a national AAI organisation) and informal (self-guided study). Topics within the formal training, across 16 h, included: AAI terminology, dog welfare, workplace considerations, zoonosis, health and safety, amongst other theory presented prior to hands-on practical training with the dog. In addition to the formal handler-dog team training, this therapist also prioritised training of the dog’s obedience, manners, and tonal conditioning. Two other therapists reported that their dogs had received general dog obedience training, conducted by external parties and reinforced by the therapists. They did not discuss the specific training activities or whether it impacted the therapy sessions, however. One therapist’s dog was engaged in agility and track/field.

All therapy dogs worked less than five days per week, most commonly seeing five clients per day for a one-hour session each. However, the current study’s methodology did not explore exactly how much of this “working time” involved active versus passive participation for each dog. The stress levels and well-being of the dogs were monitored via a variety of methods throughout the working day by all therapists. Some therapists managed stress in the dog by providing an open crate/safe space for the dog to voluntarily withdraw from the session. Another utilised the crate to remove the dog from client contact if the dog appeared tired or agitated. Others used a “time out” from contact in the session, if the dog was yawning, moving away from the client/hiding, excessively sleeping, or displaying dissatisfaction with client/task via their facial expressions. One therapist incorporated rest days (with no work) to promote well-being in their therapy dog.

#### 3.2.2. Perspectives on Canine-Assisted Speech Pathology

When asked to describe their overall experience of providing canine-assisted speech pathology services for children, most therapists (*n* = 3/5) indicated joy and satisfaction in combining their love of animals with speech pathology, according to thematic analysis of interview transcripts. For example, one therapist stated that with the dogs’ assistance, therapy “felt like home for the children and it felt like home for me.”–Therapist 4. The therapists also appreciated the creative therapy methods that could be used in these sessions, frequently brainstorming ways to meaningfully incorporate the dog to facilitate therapy goals. However, several clinicians (*n* = 3/5) also noted that managing the needs of both the dog and client during sessions could be complex: “It can be really hard to manage a dysregulated dog and a dysregulated child in the same room.”–Therapist 5; “…a great deal of multi-tasking [is required]!”–Therapist 4.

In outlining the manner in which they included the therapy dogs into their sessions, the therapists unanimously spoke of conducting structured and interactive tasks that supported therapy goals (e.g., articulation games or storytelling). The majority (*n* = 4/5) also included the dog in sessions to motivate their clients and to support emotional grounding; “…they also make things, many things a lot easier as well. Particularly emotional regulation.”–Therapist 1. Finally, the therapists specifically employed the dogs as bridges to communication, encouraging social interactions and promoting natural language use, as shown in the following quotes: “I use my therapy dog to initiate social communication. The child interacts with the dog first, then extends that skill to people.”–Therapist 2; “I treat the dog like a professional colleague, using him as an example to teach conversational turn-taking.”–Therapist 4.

The therapists also noted differences between traditional speech pathology delivery and canine-assisted sessions, although these varied. Specifically, one therapist recalled a lack of engagement opportunities without the dog and reduced creativity. Another felt that traditional therapy was less taxing. Finally, one therapist noted that therapy without the dog lacked the provision of instant rapport, excitement, and strong emotional bonds evident when the dog was present.

In terms of the influence of the dogs upon therapy, all therapists mentioned the role of the motivator, improving engagement of the client with therapy tasks, as well as encouraging the child to attend therapy. For instance, “If they don’t want to do an activity, but I make a way to include [Therapy dog], they all of a sudden want to do it.”–Therapist 5. Additionally, all therapists reported on the emotional regulation and calming influence exerted by the dog’s presence. The therapists also considered facilitation of social skills and language development to be a key influence of the therapy dog (e.g., use of prepositional phrases, storytelling). Some shortcomings of including therapy dogs, however, were also discussed by the therapists, include the need for multi-tasking, managing clients who were not comfortable around dogs to build trust and feelings of safety, and dealing with logistical issues, such as space and hygiene.

It was thought by the therapists that there were certain prerequisites for a client to be suitable for working with a therapy dog, in line with the clinic’s child suitability survey that is conducted prior to therapy. For instance, the therapists believed that children who displayed harmful or aggressive behaviour towards animals would not be suitable for canine-assisted speech pathology services. Similarly, all therapists considered that certain qualities were needed in a dog, to class them as suitable for inclusion in therapy. Namely, the dogs should be calm and adaptable, matching the energy levels of the clients but not engaging in hyperactivity. Most also felt that the dogs should be gentle and approachable, to facilitate interactions (*n* = 3/5).

The therapists were specifically quizzed about any challenges they had encountered since commencing delivery of canine-assisted speech pathology services. All responses cited the difficulties of balancing the workload with respecting the needs of the dog. For instance: “With [Therapy dog], I can’t really do back-to-back sessions like when I work alone, because I need to make sure she has time to rest and reset.”–Therapist 5. Some therapists (*n* = 3/5) also mentioned the challenges associated with training a dog whilst working as a therapist at the same time and the need to continuously adapt therapy plans based on the dog’s developmental stage. Logistic issues, such as the need for designated rest areas, distraction by other dogs, and hygiene requirements, were again mentioned by most therapists. For example, “We have multiple dogs here, which means multiple personalities… and that can be interesting to juggle.”–Therapist 2. Several therapists were also required to handle unexpected incidents involving the therapy dog, for example, minor injuries or discomfort caused to clients; “I do my best to keep her claws neat, but sometimes things happen, and it’s not fun.”–Therapist 2.

In order to advance the practice of canine-assisted speech pathology, three of five therapists stated the need for more structured training and accreditation in the canine-assisted therapy sector, official recognition of the field by professional and governing bodies, increased interdisciplinary collaboration and broader uptake of canine-assisted therapy across professions, as well as expanded research and resources. They also noted the importance of workplace infrastructure in successfully incorporating therapy dogs into a Speech Pathology clinic. Suggestions include consideration of the clinic’s layout, having sound-proof rooms and one-way glass to reduce distractions for the dog, and secure doors to prevent accidental human–animal and animal–animal interactions.

Lastly, therapists were asked if they had any final thoughts regarding their therapy dogs and/or the canine-assist speech pathology service. Thematic analysis revealed five key content themes: (1) The positive impact of therapy dogs (including upon emotional regulation, social communication, motivation, and engagement) (*n* = 5/5); (2) The challenges and extra responsibilities involved in incorporating a therapy dog into services (including the organisation and planning required, as well as dog care, and managing the dog and client simultaneously) (*n* = 4/5); (3) Therapy dogs as confidence builders (*n* = 3/5); (4) Therapy dogs providing support and comfort beyond the client (to caregivers and therapists) (*n* = 3/5); and (5) Therapy dog behaviour as a teaching tool (spontaneous learning) (*n* = 3/5).

## 4. Discussion

This study aimed to explore therapist and caregiver perspectives on canine-assisted speech pathology for children. These clients were predominantly attending therapy for speech delays/disorders, delayed language acquisition, and/or difficulties in social skills. Overall, both the caregivers and therapists believed the inclusion of therapy dogs in speech pathology sessions produced a notable improvement in the children’s mood, engagement with therapist and therapy task, rapport with the therapist, and motivation, and a decrease in stress/anxiety during therapy. The majority believed that the ability of the therapy dog to enhance the children’s motivation to attend and engage, as well as to provide emotional support and regulation, was positively related to therapy goal attainment.

These findings align closely with the previous literature exploring parent and therapist perspectives of canine-assisted interventions in other disciplines, such as occupational therapy [8,12,14]. In the current study of speech pathology services, goal attainment was perceived to be facilitated by the dog, particularly in the areas of language and social skill development. Such is supported by the results of a recent systematic review of the effectiveness of animal-assisted therapies for children and adolescents with ASD. Although the review was unable to definitively recommend animal-assisted therapies for this population, due to heterogeneity of the evidence base, a consistently positive impact was found on social domains [15]. It was noted that animals likely influenced therapy outcomes due to their roles as social facilitators, motivators, and social supports. Studies also highlighted the predictable nature of interactions with animals, rendering them less threatening and more appealing than interactions with humans [15].

The interviewed therapists considered enjoyment of interactions, and ability to bond, with dogs to be requisites for paediatric clients of canine-assisted speech pathology services. Accordingly, most caregivers noted that their children greatly enjoyed this mode of therapy and were aware of the development of a positive connection between the dog and child. Although references concerning the human–animal bond within canine-assisted speech pathology services are very limited, it is known in a broader context that secure relationships are often more readily developed with animals than with human partners, particularly among children with developmental delays [16]. Furthermore, the unconditional and non-judgmental relationships with the therapy dogs reported in the current study may have counteracted the children’s feelings of failure and learned helplessness that often result from poor task performance in therapy activities [17]. The outcomes of the current study, in fact, echo those of Hill et al. [12] in relation to canine-assisted occupational therapy. Within the latter study, parents described the relationship formed between their child and the therapy dog as a “friendship.” They believed the development of this supportive relationship created an emotionally safe space for their child, which, in turn, facilitated the rapport built between their child and the therapist [12]. Further comments from the present study’s therapists highlighted that the therapy dogs appeared to accelerate the process of building rapport with clients, particularly those whom caregivers described as being initially resistant to traditional therapeutic methods. The Speech-Language Pathologists found that establishing rapport was significantly easier, even with new clients, when the dog was present, and hypothesised that deflecting the spotlight onto the therapy dog made children less wary.

The therapists further reported that the enhanced motivation and progress seen in clients of canine-assisted speech therapy contributed to their own job satisfaction, consistent with the results of an AAT meta-analysis by Nimer and Lundahl [18]. The therapy dogs’ positive impact on client goal attainment may also help reduce the risk of job burnout among therapists [19]. Whilst the therapists enjoyed the overall experience of working alongside the therapy dogs, they also stressed the complexities involved in creating a safe, comfortable, and effective environment for all involved parties: the client, therapist, and dog. For example, it could be stressful for the therapist and therapy dog if the child became overly fixated on the dog, or if the dog were to feed off of the child’s high energy levels. It becomes the responsibility of a skilled therapist to remain calm, to intervene, and to regulate both the child’s and the dog’s behaviour, maintaining a safe therapeutic environment that is conducive to goal achievement.

Interestingly, within the previous literature discussing the benefits of including a therapy dog into interventions with paediatric clients, past research has highlighted that it was not the therapy dog alone that affected this change, but how the therapist and the therapy dog worked together as a team [12,14]. Within the present study, beyond one therapist referring to their therapy dog as a “colleague,” no parent or therapist participants made mention of the team dynamic between the therapist and their therapy dog, or how they worked together to successfully facilitate child motivation and engagement. Instead, participants focused on the impact of the dog alone, perhaps, indicating a broader rather than nuanced appreciation of the unique team dynamic between therapist and dog. This finding may also be reflective of the interview questioning not specifically mentioning the term “team.”

Further, the previous literature has emphasised that animal-assisted therapy was never designed to be used as a stand-alone intervention, but instead included as an adjunct to pre-existing, evidence-based interventions [20]. All participants within the present study discussed simple ways in which the therapy dogs were incorporated into the therapy session (e.g., including the therapy dog in games, talking to and physically interacting with the therapy dog). Therapist participants also discussed the benefits of being able to “creatively” find ways to incorporate their therapy dog into therapy activities. However, the interviews did not reveal details on exactly how the therapists incorporated their therapy dog into activities to facilitate goal achievement, nor the clinical reasoning used to determine when it was or was not appropriate to involve their therapy dogs. To ensure that canine-assisted speech therapy is delivered safely and effectively, further research is required to explore how speech therapists include their therapy dog into interventions with paediatric clients, including their decision-making process.

The interview participants provided information related to the characteristics and training of the therapy dogs. The therapy dogs had been selected for their working roles, by the therapists, largely based on temperament; specifically, for a calm, gentle, approachable, and flexible nature. Commonly, the dogs had completed obedience training, as an informal minimum requirement for inclusion in therapy practice. However, only one therapist had undergone training in AAT (including as a handler/animal team). This finding was notable in view of the typical types of interactions encountered between the children and dogs during sessions being associated with high risk (i.e., physical contact with the dog, feeding the dog, and playing with the dog were reported). It is widely acknowledged that AAT is a complex area of practice requiring additional training and experience for it to be implemented safely and effectively [5].

Whilst the therapists within the present study acknowledged these complexities in relation to managing the well-being needs of their therapy dogs, whilst also meeting the needs of their clients, it was unclear what specific steps were taken to meet these responsibilities. Specific challenges mentioned by the therapists within the present study included workload planning, time management, logistics, and multitasking, to ensure that the welfare and safety needs of the dog and child, respectively, as well as therapy goals, could be met. Participants noted that multiple therapy dogs were often present within the clinic; however, they did not elaborate on any specific organisational policies or procedures related to how these considerations were managed. The authors acknowledge that this omission may have been a shortcoming of the interview technique.

Regarding the welfare of the dogs, the therapists reported restricting the dogs’ working week to less than 5 days, with only 5 clients seen per day for approximately an hour each, during which times (before, during, and after therapy sessions) it was necessary to informally observe the dogs’ behaviour for stress monitoring purposes. Whilst research exploring the well-being of therapy dogs is limited, some work has been conducted providing recommendations to therapists. For example, within a 2024 study investigating therapy dog welfare when interacting with autistic children within canine-assisted occupational therapy sessions, several recommendations were made [21]. These included: the therapy dog handler having received training to identify and appropriately respond to their therapy dogs’ signs of stress; the therapy dog working off lead during sessions to allow them to interact with the children of their own volition; having one full rest day in between working days (i.e., maximum three days per week); as well as having active involvement of a maximum 15 min within each therapy session [21]. These recommendations are also supported by the Australian Code of Conduct for the Animal-Assisted Services Sector [22]. As the current study’s methodology did not explore exactly how much of the dogs’ “working time” comprised active versus passive engagement with clients, nor did it investigate scheduling of break or rest times, it is not possible to strictly compare the practices of the study’s clinic with those recommendations seen in the literature. However, it has been previously highlighted that therapists working without the appropriate training in animal-welfare and animal rights, as well as how to put these into practice, are at risk of not working ethically within the field, as well as posing reputational risk to the profession [20]. As such, further research is required to establish what level of training is required by speech therapists delivering canine-assisted therapy with paediatric clients to ensure they have the knowledge and skill to take the necessary steps to ensure the safety and well-being of their clients and therapy dogs. Furthermore, this research should consider the journey of the human–animal team, as they build their expertise in the field of canine-assisted therapy. Supporting guidelines could be devised to assist teams as they progress through the various stages of their careers, from novice to expert level.

While the present study offers valuable insight into the perceived benefits of canine-assisted speech therapy, there is considerable scope to expand the evidence base in ways that could meaningfully advance both research and practice. Beyond evaluating immediate outcomes, future research might investigate whether therapy dogs can fundamentally reshape children’s long-term relationship with communication, foster resilience, or shape how families engage with and support the therapeutic process. Questions also remain around what truly constitutes “best practice”—not only in terms of session design, but also in how therapists and dogs work as collaborative partners, how the welfare of animals is safeguarded, and how these models might be scaled sustainably across services. Exploring these questions has the potential to move the field from demonstrating benefits to defining an innovative and ethical framework for practice that could influence paediatric therapy more broadly. Future research should also consider observational research on therapy animal welfare, which is currently limited. Studying whether there are breed differences regarding animal behaviour or child preference in this context would also be worthwhile.

Longitudinal studies could provide valuable insights into the sustainability of therapeutic gains and the potential for lasting improvements in communication skills [23]. Examining the effects of therapy dogs across more diverse therapeutic settings and paediatric populations is essential to determine the generalisability and scalability of these novel interventions [20]. Additionally, to enhance the quality of the services provided, as well as the welfare of all involved, further research should look to explore and understand the training required of speech therapists delivering canine-assisted therapy, as well as their clinical reasoning used when deciding to implement this unique intervention.

### Limitations

While this study provides valuable insights into the therapist and caregiver perspectives of canine-assisted speech therapy, several limitations must be acknowledged. Firstly, canine-assisted speech therapy is a relatively new field, and the analyses included only five therapists. This small sample size may limit the generalisability of the results, as it captures a narrow spectrum of experiences and perspectives. Furthermore, all participating therapists were affiliated with the same clinic, and the parents interviewed were clients of this clinic. This homogeneity may introduce selection bias and restrict the diversity of viewpoints. Consequently, the findings may not be representative of other clinical settings or broader populations, and should be interpreted with this in mind.

## 5. Summary and Conclusions

This study revealed that therapy dogs function as essential facilitators in paediatric speech-language therapy rather than mere supplements. The dogs were perceived to directly enhance communication development by serving as catalysts for spontaneous language, reducing anxiety around speaking tasks, and enabling children to take communicative risks in a non-judgmental environment. Their presence was felt to strengthen goal attainment across expressive and receptive language, social communication, and pragmatic skills, with children demonstrating increased verbal output, longer sustained interactions, and more frequent initiations. The dogs’ ability to counteract avoidance behaviours and learned helplessness proved particularly valuable for children who typically struggle with traditional speech tasks.

Beyond motivational benefits, therapy dogs significantly accelerated rapport-building and created optimal conditions for natural language emergence. By redirecting attention from the child to the dog, clinicians observed that even hesitant or resistant children engaged more readily in foundational speech therapy activities including turn-taking, commenting, and descriptive language. The cumulative effects of enhanced motivation, richer verbal interactions, stronger social-pragmatic engagement, and expedited therapeutic relationships demonstrate why dogs hold distinctive relevance in speech therapy contexts, supporting their thoughtful integration into paediatric speech-language intervention practices.

## Figures and Tables

**Table 1:** Types of speech pathology services received by 42 children attending canine-assisted sessions. (Note: N is greater than 42, as some children were treated for more than one condition.).

Condition	N
Speech delay	20
Language delay	16
Speech disorder	12
Other—Autism	8
Fluency	8
Language disorder	7
Social pragmatics	4
Voice	2
Literacy	2
Other—Intellectual disability	1
Other—ADHD	1

**Table 2:** Types of interactions with therapy dog during canine-assisted speech pathology sessions (*n* = 42).

Interaction	Yes Responses (*n*)	*n*/42 (%)
Physical touch (e.g., pat, hug)	36	85.7
Give a treat	32	76.2
Play with a toy	29	69.0
Talking to	29	69.0
Play a game	27	64.3
Watching	27	64.3
Other—teaching tricks	2	4.8
Other—nothing, dog sleeps, quiet time together	2	4.8

**Table 3:** Caregivers’ perceptions of dog influence on their children’s therapy sessions (*n* = 42).

Influence	Yes (*n*)	*n*/42 (%)
Improved mood	30	71.4
Decreased stress and anxiety	29	69.0
Improved engagement in activities	26	61.9
Improved motivation	26	61.9
Improved engagement/rapport with therapist	25	59.5
Improved attention	21	50.0
Improved perseverance with difficult tasks	16	38.1
No influence	3	7.1
Other—including negative influences	0	0.0

**Table 4:** Caregivers’ perceptions of the influence of the therapy dog on their children’s achievement of speech therapy goals (*n* = 42).

Theme	Responses (*n*)	Description
Motivation and Willingness to Attend	14	Increased excitement, willingness to attend and participate; e.g., ”She so looks forward to her sessions…”
Emotional Support and Calming	13	Dog helps regulate emotions, calms anxiety, reduces stress; e.g., “Put my child at ease from the first session. It seemed to remove any reservations my child had about being in a new environment.”
Social Confidence and Communication	9	Encourages speaking, reduces pressure, builds confidence; e.g., “He provides her with the chance to take the focus off herself… gives her more confidence.”
Engagement and Focus	7	Helps maintain attention and engage more effectively; e.g., “I don’t believe that our child would get through a full hour of speech therapy without the dog being present.”
Goal Progress	5	Perceived improvement in achieving speech therapy goals; e.g., “Noticeable improvement with child’s goals compared to when seeing a speech therapist without therapy dog.”
Routine and Structure	2	Dog helps establish predictable structure during sessions; e.g., “The dog helps my child recognise what activities we might be doing… sense of routine and expectation.”
Uncertainty/Neutral Responses	4	Responses expressing uncertainty or limited impact noted; e.g., “Unsure.”

## Data Availability

Due to concerns regarding anonymity of participants, de-identified data is available from the authors upon request only.

## Appendix A

**Table A1:** Therapist Interview Questions.

1. How many years have you been practicing as a Speech Pathologist? 2. How many years have you been working with a canine in delivering speech pathology services? 3. Have you completed any training in animal-assisted therapy and, if so, what did this involve? 4. Do you own your current therapy dog? 5. How many years have you been working with your current therapy dog? 6. From where did you purchase/adopt your therapy dog? 7. Has your therapy dog completed any particular training and, if so, what did this involve? 8. What breed is your therapy dog? 9. How old is your therapy dog? 10. Is your therapy dog male or female? 11. How many days a week does your therapy dog work? 12. How many clients does your therapy dog see each day? 13. How long is each canine-assisted therapy session? 14. Do you monitor for signs of stress in your therapy dog and, if so, how? 15. Is there anything else you would like to tell us about your therapy dog? 16. Can you please tell me about your experience of working as a Speech Pathologist who delivers canine-assisted therapy to children? 17. How do you think the presence of your therapy dog influences the children? (Benefits and shortcomings) 18. How do you think the presence of your therapy dog influences you when working with children? (Benefits and shortcomings) 19. How do you think this is different from not working with a therapy dog? 20. Would you please be able to briefly outline an example of what a session would look like with you and your therapy dog? How do you incorporate your dog into your speech pathology interventions? 21. Do you think that there are any specific prerequisites for a client to be suitable for working with a therapy dog? 22. Do you think that there are any specific qualities/temperaments that make a dog suitable for work as a therapy dog? 23. Have you been faced with any challenges since starting practice as a canine-assisted Speech Pathologist? 24. What do you feel is needed for the practice of canine-assisted Speech Pathology to move forward? 25. Is there anything else you would like to tell us about your thoughts on canine-assisted Speech Pathology services?

## Appendix B

**Table A2:** Caregiver Survey Questions.

1. For how long has your child been receiving canine-assisted speech pathology services? 2. For which conditions has your child been receiving therapy? 3. What are your child’s speech therapy goals? * 4. Is there anything else you would like us to know about your child? * 5. How do you believe your child enjoyed the therapy sessions when the therapy dog was present? 6. What words would you use to describe your child’s relationship with the therapy dog? * 7. In what ways do you believe the therapy dog influences your child’s therapy sessions? * 8. In what ways do you believe the presence of the therapy dog has influenced your child’s achievement of their speech therapy goals? * 9. Do you think the therapy sessions with the therapy dog present are different for your child, when compared to when the therapy dog is not present? * 10. What type of interaction does your child engage in with the therapy dog within their sessions? 11. Does your child have, or has your child had, a pet dog at home? 12. Are there ways in which you believe Speech Pathology services with therapy dogs could be improved? * 13. Is there anything else you would like to tell us about your thoughts on your child’s experience in canine-assisted speech pathology? *

* Indicates an open-ended response format.
